# A Scientometric Exploration of the Trends, Distribution, and Collaborative Networks of Dental Fluorosis Research in India

**DOI:** 10.1097/PHH.0000000000002430

**Published:** 2026-08-12

**Authors:** Sree Keerthi Vavintaparthi, Sibyl Siluvai, Raghul Gandhi Venkatesan, Indumathi Kariannan Partheeban, Krishnaprakash Govindaraj, Saravanan Anbukumari Vadivelu

**Affiliations:** **Author Affiliations:** Intern, Department of Public Health Dentistry, SRM Kattankulathur Dental College and Hospital, SRM Institute of Science and Technology, Kattankulathur, Chengalpattu District, Tamil Nadu, India, (Vavintaparthi); Professor & HOD in charge, Department of Public Health Dentistry, SRM Kattankulathur Dental College and Hospital, Faculty of Medicine and Health Sciences, SRM Institute of Science and Technology, Kattankulathur, Chengalpattu District, Tamil Nadu, India (Siluvai), Centre for Statistics, SRM Institute of Science and Technology, Kattankulathur, Chengalpattu District, Tamil Nadu, India (Venkatesan), Assistant Professor, Department of Public Health Dentistry, SRM Kattankulathur Dental College and Hospital, Faculty of Medicine and Health Sciences, SRM Institute of Science and Technology, Kattankulathur, Chengalpattu District, Tamil Nadu, India (Kariannan Partheeban); Assistant Professor, Department of Public Health Dentistry, SRM Kattankulathur Dental College and Hospital, Faculty of Medicine and Health Sciences, SRM Institute of Science and Technology, Kattankulathur, Chengalpattu District, Tamil Nadu, India (Govindaraj); Professor & HOD, Department of Periodontology, SRM Kattankulathur Dental College and Hospital, Faculty of Medicine and Health Sciences, SRM Institute of Science and Technology, Kattankulathur, Chengalpattu District, Tamil Nadu, India (Anbukumari Vadivelu).

**Keywords:** dental fluorosis, India, prevalence, risk factors, water fluoride levels

## Abstract

**Background:**

Dental fluorosis is a developmental disturbance of dental enamel caused by successive exposures to high fluoride concentrations during tooth development. Approximately 65% of India's rural population is directly exposed to fluoride-related health problems, according to a report from UNICEF.

**Objective:**

To perform a scientometric analysis of dental fluorosis research in India with a focus on mapping research trends, geographic distribution, collaborative networks, and thematic key insights, while reviewing and comparing the reported prevalence and associated risk factors from the literature.

**Research design:**

A descriptive scientometric study was conducted to evaluate the research landscape on dental fluorosis in India. Articles were retrieved from databases such as “PubMed, Scopus, EBSCO-HOST” and analyzed using tools like VOS viewer and R software to map the geographic distribution, keyword patterns, year-wise trend, and co-authorship networks. In addition, reported prevalence and risk factors are evaluated and reviewed.

**Findings:**

The analysis expressed a rising trend in dental fluorosis research, with higher concentrations of studies in fewer parts of India. Common keywords concentrated more on fluoride exposure and its epidemiology, where co-authorship networks indicated limited interinstitutional collaboration. Reviewed studies demonstrated varying prevalence rates across India and associated risk factors, which showed inconsistency throughout the derived studies.

**Implications:**

This scientometric overview refers to an increasing but unbalanced base of dental fluorosis studies in India. It forms the basis of identifying gaps in the field of research and directing further studies to aid in the reduction of fluorosis prevalence in India.

## Introduction

The mineral fluoride is found naturally in various foods and is available as a dietary supplement. Fluoride is the most effective agent in preventing dental caries, according to the World Health Organization's Oral Health Report (2003).^1^ However, fluoride has 2 drawbacks: it can prevent cavities if taken in the right doses (0.7–1.2 ppm), but it can disrupt the body's calcified tissues, leading to fluorosis if it exceeds the ideal limit.^2^

The developmental disruption of dental enamel, known as dental fluorosis, is brought on by repeated exposures to excessive fluoride concentrations during tooth formation. As a result, enamel becomes more porous and has less mineral content. The World Health Organization (WHO) states that horizontal streaks and bilateral symmetrical lesions on the surface of the dental enamel are signs of dental fluorosis. In milder grades, fine, opaque, white lines can develop into brown spots or depressions with corrosion at the most severe levels. It is brought on by consuming too much fluoride over an extended period during the tooth-formation process, mostly from a combination of sources like toothpaste and the public water supply.^3^ Few studies have been published in the literature that examine the local risk factors for dental fluorosis and link fluoride content in water with fluorosis.

India is one of more than 24 nations worldwide where dental fluorosis is common. Many places in India have high levels of fluoride minerals. Approximately 65% of India's rural population is directly exposed to fluoride-related health problems, according to a report from UNICEF. The fluorosis issue is especially serious in regions like Andhra Pradesh, Tamil Nadu, Rajasthan, Punjab, Bihar, Uttar Pradesh, Madhya Pradesh, and West Bengal, where the drinking water fluoride content is much greater than the permissible threshold. By synthesizing existing research, this study aims to identify trends, assess the burden of the condition, and highlight contributing factors, such as fluoride levels in water, dietary habits, and socioeconomic conditions. This analysis seeks to inform public health policies and strategies to mitigate the impact of dental fluorosis on affected populations.

This study aims to use scientometric analysis to answer the following research questions:

1. Which geographic locations in India have been most studied for dental fluorosis, and where are the research gaps?
2. How has the publication output on dental fluorosis in India evolved beyond 2010 till 2024?
3. Who are the most influential and collaborative authors in dental fluorosis research in India, based on co-authorship and total link strength?
4. Which keywords are most commonly used in dental fluorosis research conducted in India, and what subject areas do they represent?

A scientometric analysis is characterized as a “quantitative study of science, science policy, and communication in science.” Examining citation links, assessing the research effect, and examining the impact of academic journals and research institutes in a particular field of study are all included in the scientometric analysis. In this proposed method, a hybrid approach of 2 main strategies will be used, namely scientometric analysis (bibliometrics), which incorporates monitoring the trends of research, its geographical distribution, collaboration patterns, and classifications through the use of VOSviewer software, and systematic reviews, whereby the results of prevalence studies conducted in India will be summarized under PRISMA 2020 guidelines. The methodology, findings, and interpretations, a discussion of various issues and considerations that are elaborated to answer the research questions, and the conclusion are the 4 sections that make up this article.

## Literature review

In a recent analysis conducted by Hitesh Chander Mittal et al, the objective was to ascertain the distribution of dental fluorosis by geographic area, water fluoride level, and severity in India. The outcome of their study showed areas with water fluoride levels above 1 ppm had a prevalence that was more than 3 times higher than those with levels below 1 ppm (50.3% vs. 15.1%). Furthermore, higher prevalences were observed in men (40.7%), communal settings (40.7%), rural individuals (38.1%), and the western Indian region (43.9%).^4^

In another study conducted, the researchers sought to ascertain the fluoride content of the selected study area from a range of sources, such as cow's milk, cooked rice, drinking water, and green leafy vegetables. The percentage of fluorosis cases was estimated using a clinical survey. The computed community fluorosis index values for each of the 22 communities exceeded the recognized index value. The study discovered that, among all the sources, drinking water gives a significant quantity of fluoride, regardless of age. Residents of South India, where food patterns are similar, are therefore advised to drink water with less fluoride to lessen the negative effects of fluoride.^5^

A review conducted by Shaz Ahmed et al examined the most recent studies on the potential harm that excessive fluoride may cause to a variety of animals. It concluded that fluoride-contaminated water is particularly dangerous for human health and requires very effective water and environmental management. Because fluoride is produced into the environment by human activities and most problems are caused by ingesting fluoride from natural sources, it is a major health concern. Fluoride must be removed by the UNSDG for the Clean Drinking Water guideline, which provides a workable, affordable, and effective method. This method avoids the fundamental problems. It has been noted that it is challenging to develop precise and meaningful criteria when there are few data. Few researchers can focus on developing particular solutions because they combine many tactics to produce synergistic approaches for fluoride elimination.^6^

A cross-sectional study was done by Anand Verma et al.^7^ The purpose of this study was to determine the factors linked to dental fluorosis in Kolar taluka, Karnataka, India, and to measure the prevalence of dental fluorosis among adolescents enrolled in school. This study concluded that about two-thirds of students had dental fluorosis, which was quite common, particularly in government schools and among long-term local inhabitants. To prevent fluorosis, study communities should consider health education and community awareness campaigns in addition to establishing defluoridation plants or offering training for home-based defluoridation techniques.

Another study by Sandeep Kumar et al^8^ examined the dental fluorosis prevalence and risk factors in India's early teens. In total, 800 teenage schoolchildren were chosen from the Jhabua and Dhar districts of Madhya Pradesh, India, for this cross-sectional study. The kids ranged in age from 12 to 15 years. To estimate the amount of fluoride in the water, samples were taken from each zone and sent to a laboratory. The Dean index was used to evaluate dental fluorosis. In early teens, dental fluorosis was shown to be 40.5% prevalent overall. The Jhabua district had a 45% prevalence of dental fluorosis, whereas the Dhar district had a 36% prevalence. The water storage technique was revealed to be the second-best predictor of dental fluorosis, after water fluoride levels.

Punitha et al^9^ conducted a study in Kancheepuram, a nonendemic region of Tamil Nadu. In a cross-sectional study, 348 children between the ages of 7 and 15 years were assessed for fluorosis and used the Dean's Fluorosis Index to grade the severity of the condition. In total, 19.2% of people had dental fluorosis (CI: 15.1–23.3). Fluoride levels in drinking water sources were found to be as high as 2.2 mg/L. Fluorosis was shown to be significantly correlated (*P* < .001) with high fluoride levels. It was noted that the water was not routinely tested for purity. There are not many rural areas in this district with high fluoride levels in their groundwater. Finding a different source of water should be a priority, and above all, the quality of drinkable water should be regularly assessed.

## Research Methodology

- Research Question: The research question was framed in PCC format (Table 1).
- Protocol and Registration: This review protocol is registered in PROSPERO.

**TABLE 1:** PCC Format-based Description

PCC Format	Description
(P)—Population	Indian research papers on dental fluorosis
(C)—Concept	Scientometric trends: regional distribution, link strength, authorship networks, and keywords
(C)—Context	Databases including scientific papers from 2010 to 2024

The systematic review was conducted using PRISMA guidelines (2020 criteria; Figure 1).^10^

**FIGURE 1:**
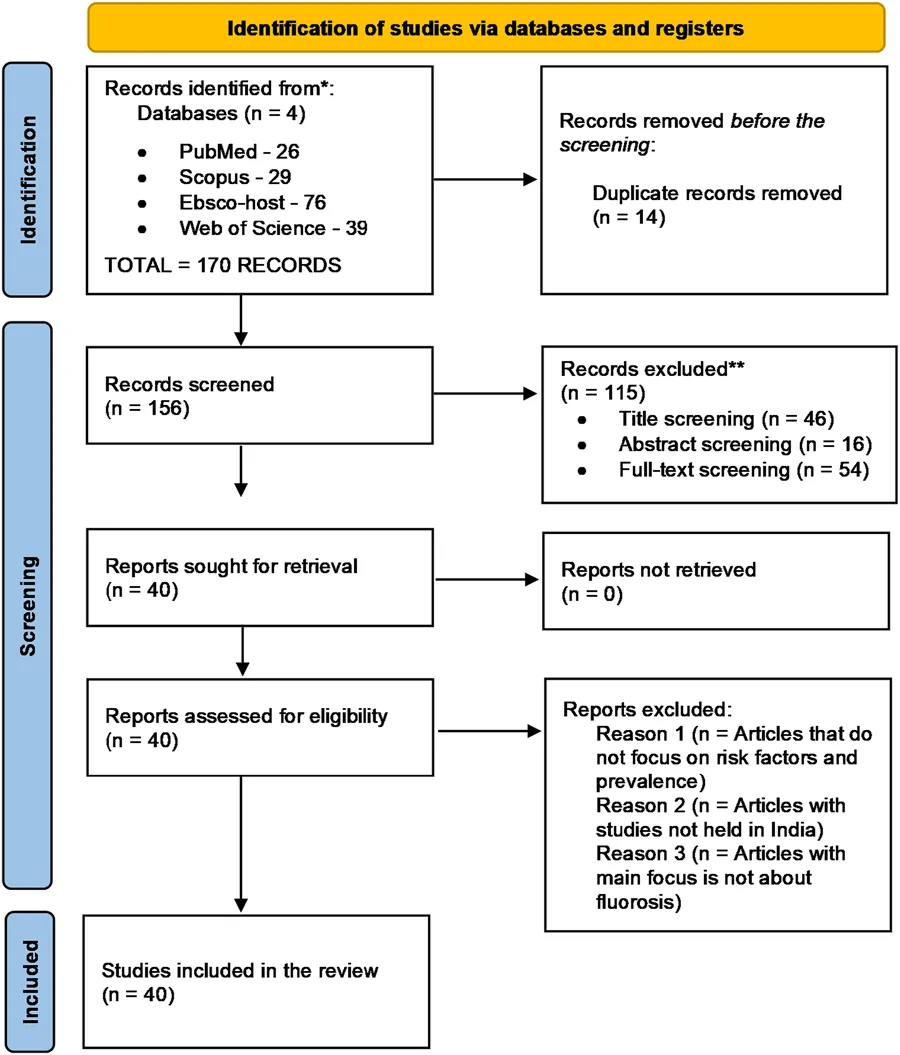
Results of search presented in PRISMA flow-chart (2020).

### Eligibility criteria

- Inclusion criteria: Recent scientific articles on dental fluorosis were generally accepted.
  - Research papers published between 2010 and 2024
  - Articles that focused on fluoride use, dental fluorosis causes, risk factors, and prevalence
  - Articles with diagnostic criteria using Dean's Fluorosis Index
  - Articles concentrating on fluorosis studies held in India
  - Articles that included original research studies, such as experimental, observational, clinical trials, or case–control studies, to provide robust and reliable evidence on dental fluorosis.
- Exclusion criteria: Studies completed before the year 2010 were excluded from the evaluation.
  - Articles that did not specifically focus on fluoride use, dental fluorosis, related etiology, risk factors, and prevalence were excluded.
  - Studies in other countries other than India are excluded.
  - Articles using diagnostic criteria other than Dean's Fluorosis Index.
  - Any review articles are excluded.

### Search strategy

Different keywords were used in different databases, such as dental fluorosis, fluoride levels, prevalence, risk factors, and India. Articles are extracted from PubMed, Scopus, EBSCOhost, and Web of Science. Abstracts were matched with titles to identify duplicates and were removed. A query with the Boolean expression “AND” is used to search databases. (((prevalence) AND (“risk factors”)) AND (“dental fluorosis”)) AND (India) was the search query.

### Study selection

A de-duplication process was carried out to remove duplicates. According to the pre-existing criteria, the articles have been examined by investigators based on their titles and abstracts. When the title or abstract was inadequate, the choice to exclude the article was made after reading the full text. Screening of the full text of all the relevant articles that seemed to meet the inclusion criteria was done. Any conflicts between the researchers were resolved through evidence-based discussion.

### Data extraction and data synthesis

As the full-text eligible articles are confirmed, data extraction sheet is created which includes: “Title, author name, year of publication, study location, study design, population setting, sample size, age range, gender distribution, diagnostic criteria, prevalence, severity, source of water fluoride, water fluoride levels, additional risk factors, duration of exposure, dietary habits, socioeconomic status, environmental factors.”

## Scientometric Analysis

Scientometric analysis is a quantitative examination of scientific research. In addition to evaluating the impact of research and examining citation linkages, it is a technique for mapping a specific field of knowledge using patterns extracted from the academic database. A manual literature review is still biased and only permits subjective interpretation, even though it can offer a comprehensive overview of a particular topic of research. To visualize and map the knowledge field, the scientometric technique was used in this work to examine the prevalence and risk factors of dental fluorosis. The scientometric method uses bibliometric data to identify research subjects and build a network model. This review study used the title, abstract, and keywords to do a bibliometric search for a comprehensive literature review on construction project complexity. The following studies were performed to uncover the study pattern: keyword co-occurrence analysis, author co-citation analysis, and document co-citation and clustering analysis. Author co-citation and keyword co-occurrence analyses provide a general overview of the study field before clustering analysis.

### State-wise distribution of publications and emerging gaps

The choropleth map provides a visually integrated scientometric dataset, indicating the density of scholarly interest in dental fluorosis by Indian states. Andhra Pradesh and Tamil Nadu, historically known to have high levels of fluoride in groundwater, have been targeted with significant scholarly interest, as many as 5–6 papers, conceivably because of both public health concerns and institutional initiatives in dental and environmental health studies. Karnataka and Maharashtra also reflect significant research participation, perhaps because of local research and enhanced access to fluoride monitoring equipment. Central and northern states such as Madhya Pradesh, Rajasthan, and Uttar Pradesh reflect moderate research coverage, although those also report areas affected by fluorosis. Himachal Pradesh, Jammu & Kashmir, and Chhattisgarh, although with little contribution, can reflect emerging awareness or isolated localized research. It should be noted that the number of publications does not represent the disease prevalence but rather the research potential and interest. States that produce more publications do not necessarily indicate higher prevalence rates of dental fluorosis but might rather have more advanced facilities for dental research, research infrastructure, or available funds (Figure 2).

**FIGURE 2:**
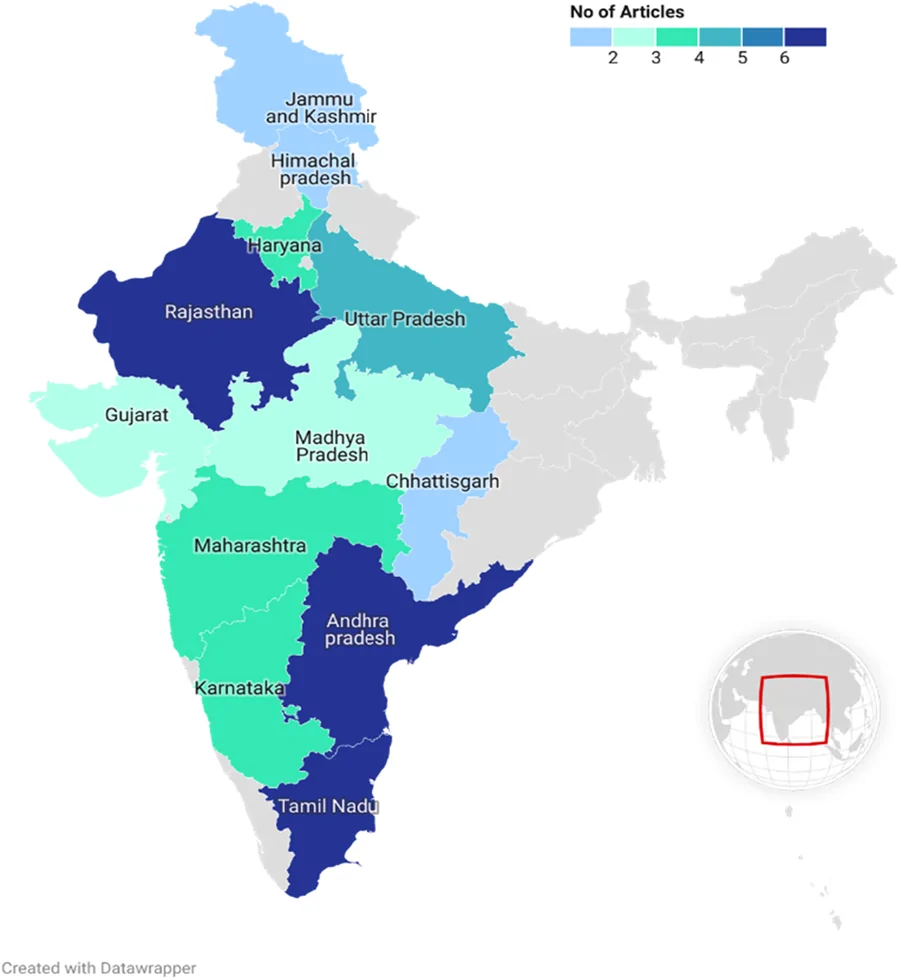
Geographic visualization of research publication on dental fluorosis in India.

What is striking, however, is the total absence of academic information from many states and Union Territories like Bihar, Odisha, West Bengal, Jharkhand, Punjab, and the entire Northeast, although many of these regions have evidenced fluoride contamination in rural water resources in government or nonacademic reports. This stark gap indicates an actual research deficit that does not necessarily express actual disease prevalence. In addition, the uneven distribution may be reinforced by state subsidies, lack of interinstitutional collaboration, or weak surveillance systems. The trend further implies a future urban–rural gap in research, where research leans toward areas with established academic networks, and faraway high-risk areas are not represented. Therefore, there is an urgent need to increase equitable research activity throughout the nation to enable inclusive health policy interventions and efficient fluoride mitigation efforts. The strengthening of cooperation among dental institutions, public health departments, and environmental departments in all Indian states would bridge these gaps.

### Temporal distributions and year-wise analysis of publication patterns on dental fluorosis

The trend of published papers on dental fluorosis in India from the years 2011 to 2024 can be seen in the bar chart (Figure 3). The highest number of publications was seen in 2013, when 7 articles were produced, the highest research interest in that year. Thereafter, peak years in 2017 and 2023 were also recorded, each with 5 articles, signifying random peaks in scholarly interest in the subject. Years such as 2012, 2014, 2016, 2020, and 2022 saw moderate activity in research with 3 or 4 publications. Conversely, years such as 2015, 2019, 2021, and 2024 had fewer publications, whereas a few years seem to have zero or insignificant research. The data show an uninterrupted but nonlinear trend in the interest for dental fluorosis research, possibly because of shifting public health priorities, local fluoride issues, or policy-led funding availability. Increased publications after 2020 can also represent a bounce back in research activity after interference by the COVID-19 pandemic. Furthermore, the increase in 2023 is consistent with increasing interest in fluorosis research, potentially fueled by improvements in technology and surveillance in endemic areas. This longitudinal investigation provides significant insight into the trend of research interest and highlights the importance of continued and geographically dispersed academic interest in dental fluorosis in response to both established and emerging public oral health concerns. In addition, production in the early part of the decade is occasional, indicating a low level of awareness or concern about dental fluorosis as a public health issue during that period. Once again, the relatively lower number of articles in 2024 may be the result of data cut-off or research yet to be published. This mapping of timelines identifies the productive research timeframes and gaps where research needs to be conducted to achieve complete national coverage. As one of the most endemic fluorosis-affected countries, prolonged surveillance of publication trends will assist in assessing the effectiveness of current fluoride abatement efforts. Systematic examination of such trends can aid in directing funding priorities, policy, and research agendas for clinical and community oral health.

**FIGURE 3:**
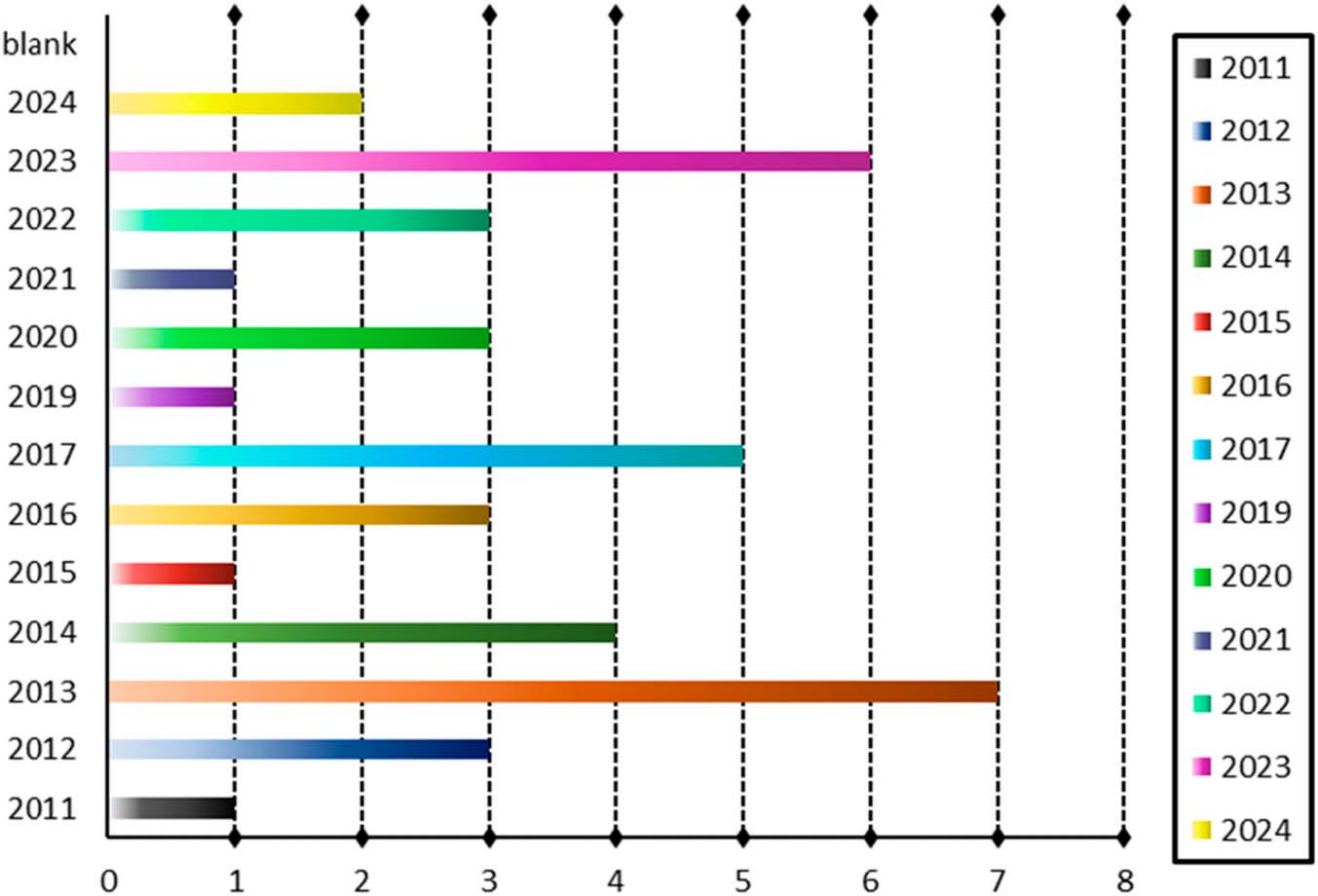
Year-wise distribution of dental fluorosis publications in India (2010–2024).

### Author collaboration analysis based on co-occurrence mapping

#### Visualizing collaborative strengths among authors in dental fluorosis research

The co-occurrence of authors within the bibliometric dataset that was input into VOSviewer presents some useful insights about the degree to which collaboration exists in the study of dental fluorosis in India (Figure 4). Initially, there were 169 unique authors in the dataset. A cut-off point for author collaboration based on the minimum number of documents an author contributed was set at zero, considering the small size of the study population (n = 40). If a more stringent cut-off point were applied, such as one where the minimum number of documents an author contributed is 2, the network becomes meaningless in the context of the disease-specific and country-specific research corpus because the vast majority of researchers would be filtered out. The connection between the authors in the bibliometric data analyzed using VOSviewer reveals some important lessons regarding the degree of collaboration among scholars researching dental fluorosis in India. There were a total of 169 unique authors included in the database. The threshold set for the minimum number of documents published by an author to consider him/her part of author collaboration was zero because of the smaller size of the data (n = 40). But had the threshold been raised to 2 documents per author, then the network created would not have served any purpose concerning the particular disease and its prevalence in a certain country.

**FIGURE 4:**
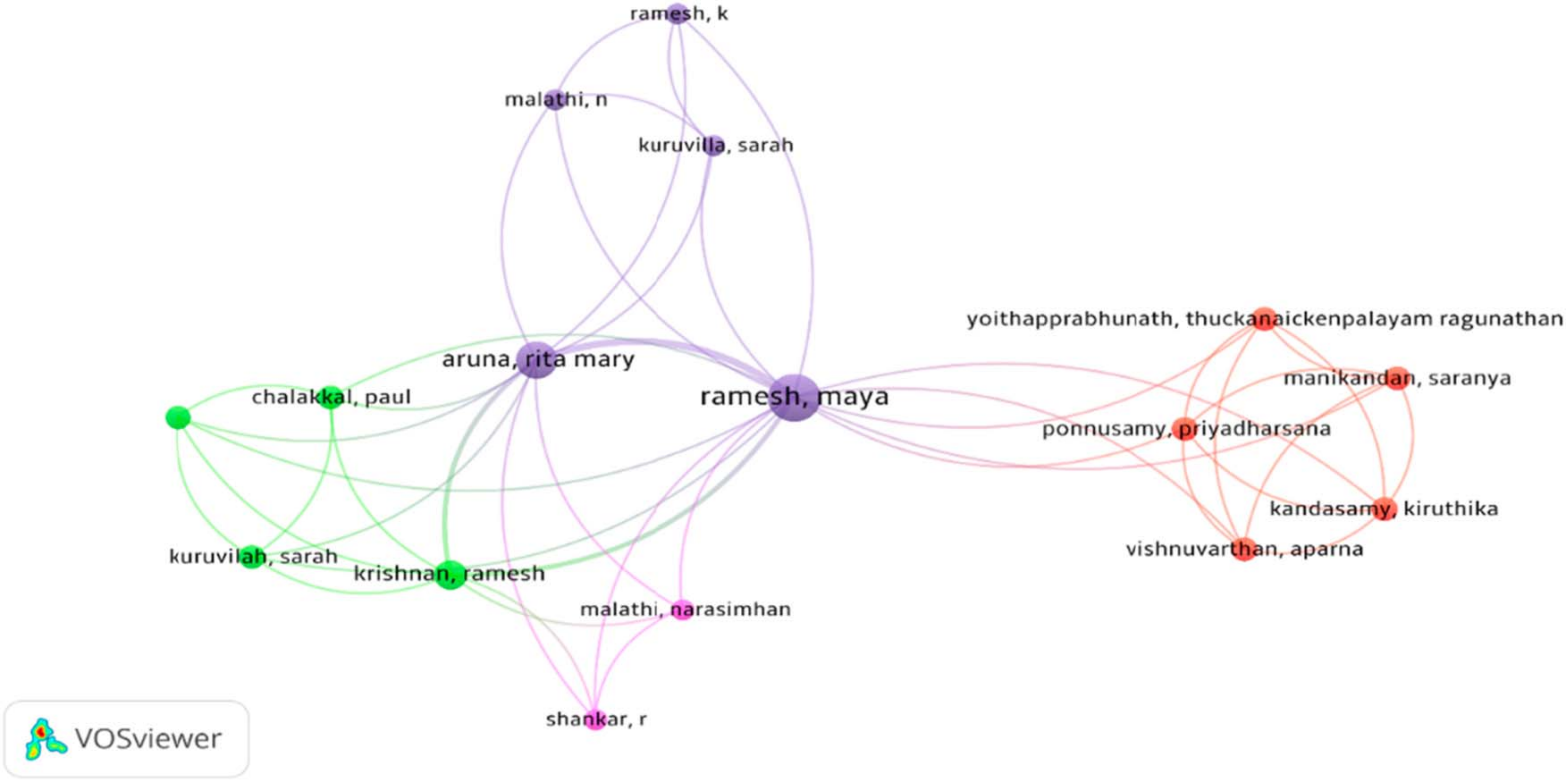
Co-authorship visualization of authors in dental fluorosis research (VOS viewer output).

#### Quantitative representation of author productivity and conductivity

The following tabulated data also clarifies the co-authorship network on the basis of the rank of authors in the overall strength of links and documents (Figure 5). Ramesh, Maya comes first with 4 papers and link strength 18, followed by Aruna, Rita Mary (3 papers, link strength 13), and others like Bala, Kumud, and Dwivedi, Supriya being behind with a link strength of 11. Rather surprisingly, even lower publication contributors like Aceituno-Antezana, Oscar, and Astudillo-Rozas, Wilson (1 paper each) have a good link strength (7), indicating high-impact collaboration. These metric quantifications underlie the visual graph and provide the foundation for objective interconnectivity and research impact analysis. The data visualization and extraction with VOSviewer not only identify the centrally positioned researchers in academic collaboration but also suggest likely singleton or gap contributors who are potentially more integrated into communities.

**FIGURE 5:**
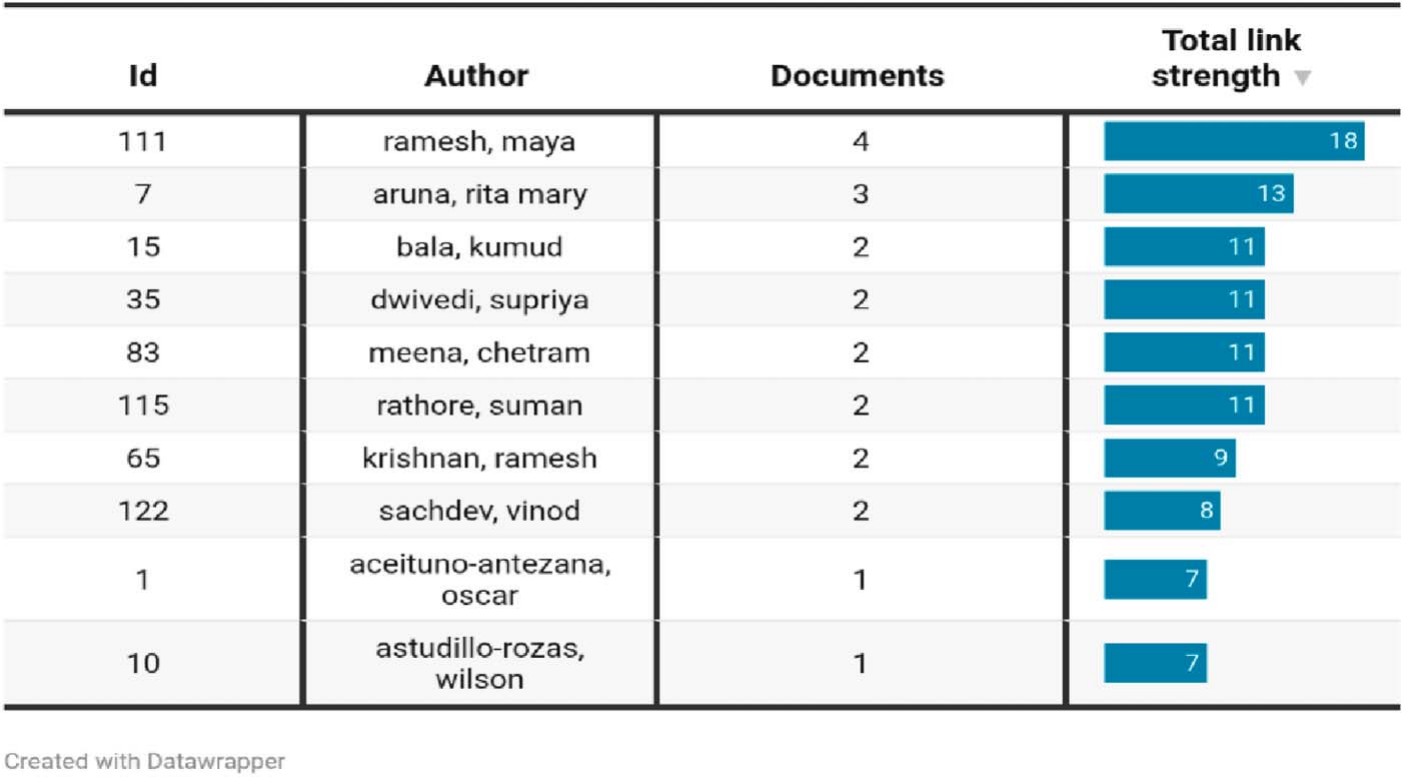
Top authors by document count and total link strength in co-authorship analysis.

#### Keyword co-occurrence mapping and thematic clustering in dental fluorosis research

In the first diagram, created using VOSviewer software, a co-occurrence network map illustrates the relationships among the significant words extracted from the titles of the selected articles on fluorosis. The node at the bottom, “fluorosis,” is connected with terms such as “district,” “prevalence,” “sectional study,” and Indian states such as “Tamil Nadu,” “Rajasthan,” and “Gujarat,” which reflect geographical prevalence and study interest. Color-coded clusters mirror thematic groupings, which facilitate the identification of concepts and geographic regions of concern. For instance, a cluster brings together “Tamil Nadu,” “district,” and “fluoride endemic” and involves regional case studies. A weakness in this visualization is that it depends on exact keyword matches, which may overlook semantic differences or contextually appropriate unquoted terms. Second, it is restricted to looking only at the title words and not the whole text of the articles (Figures 6 and 7).

**FIGURE 6:**
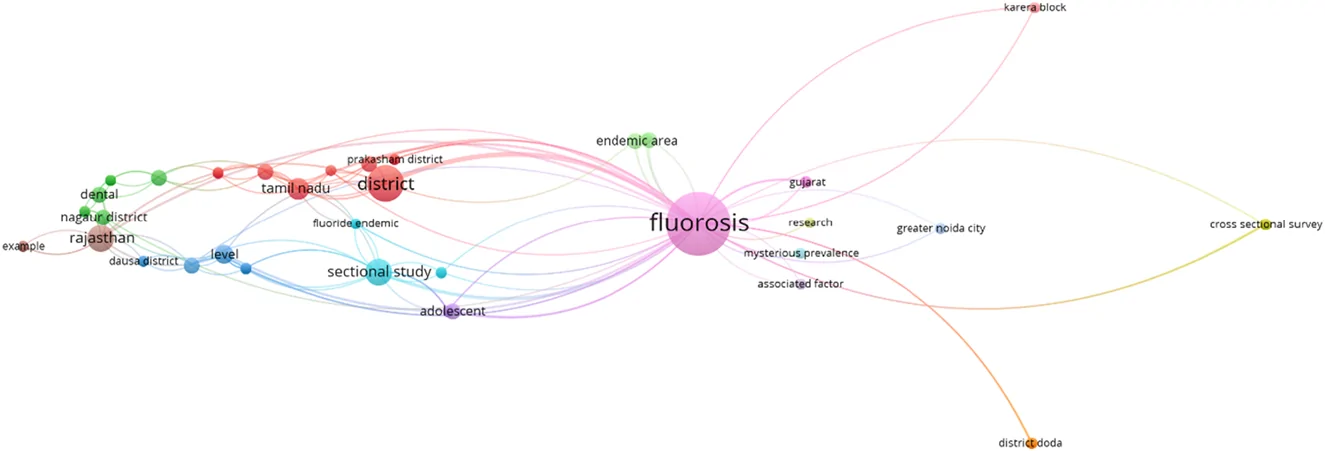
Keyword co-occurrence network of fluorosis-related research using VOSviewer.

**FIGURE 7:**
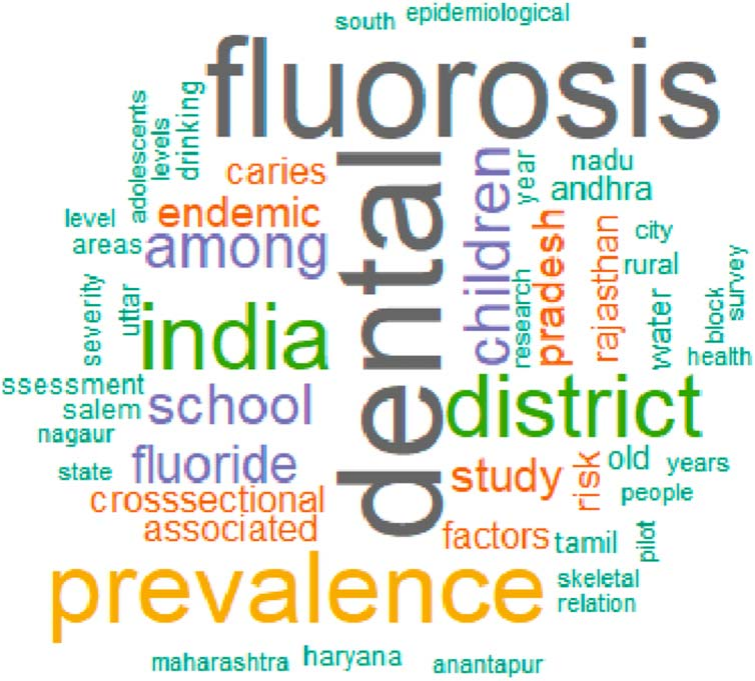
Word cloud visualization of frequently occurring keywords in fluorosis studies generated in R.

The second chart, which is an R program word cloud, also shows keywords from the titles of the included studies. The font size used gets bigger with higher frequencies of use. Dominant words are “fluorosis,” “dental,” “prevalence,” “district,” and “India,” favoring the thematic focus. It effectively presents themes of contemporary scholarship graphically in an easily understandable way so that the most common themes of investigation are easily visible at a glance. Although graphically striking, word clouds are unable to reflect the structural detail of co-occurrence maps since they do not represent inter-relationships between terms. Frequency presentation also hides context and is not able to tell apart consequential repetition from coincidental repetition. These, as a pair, offer complementary data but are limited by data level (merely titles) and lack of additional semantic examination (Tables 2 and 3).

**TABLE 2:** Study-Based Data on Fluorosis Prevalence and Water Fluoride Across India

Author	Year	Location	Prevalance	Water Fluoride Levels
Mahantesha et al^11^	2016	Karnataka	47.61%	0.63 ppm
Sirigala et al^12^	2023	Andhra Pradesh	44.05%	1.5–4.2 ppm
Patel et al^13^	2023	Gujarat	4.29%	1.25 ppm
Sandeep Kumar^8^	2020	Madhya Pradesh	40.5%	1.27 ppm
Choubisa and Choubisa^14^	2023	Rajasthan	59.0%	0.1–21.6 ppm
Aggarwal et al^15^	2020	Gujarat	93.70%	Not specifically quantified
García-Escobar et al^16^	2021	Uttar Pradesh	94.60%	1.71 ppm
Singh et al^17^	2022	Andhra Pradesh	69.9%	1.86 ppm
Patel et al^18^	2020	Uttar Pradesh	59.30%	Not specifically quantified
Sukhabogi et al^19^	2014	Andhra Pradesh	76.8%	Not specifically quantified
Verma et al^7^	2017	Karnataka	64.5%	1.4 ppm
Arif et al^20^	2013	Rajasthan	69.40%	0.5–8.5 ppm
Narayanamurthy and Santhuram^21^	2013	Karnataka	31.05%	>1.0 mg/L in 5 villages:
Naidu et al^22^	2013	Andhra Pradesh	82.04%	Not specifically quantified
Chauhan et al^23^	2012	Himachal Pradesh	4.10%	<0.2–6.5 ppm
Meena et al^24^	2017	Rajasthan	35.33%	5.4 ppm
Ramesh et al^25^	2014	Tamil Nadu	30.8%	>1.0 ppm
Shekar et al^26^	2012	Andhra Pradesh	40.80%	Not specifically quantified
Bhalla et al^27^	2015	Uttar Pradesh	18%	Not specifically quantified
Varsha and Ragini^28^	2013	Maharashtra	78%	Range: 0.53 ppm–5.0 ppm
Bhagat P^29^	2019	Chhattisgarh	14.30%	0.5–6 ppm
Reddy et al^30^	2016	Andhra Pradesh	49%	3.22 ppm
Atram Harish et al^31^	2017	Maharashtra	79.20%	Within permissible limits
Suhirtha and Subramanian^32^	2023	Tamil Nadu	51.1%	1.45 ppm
Maya et al^33^	2016	Tamil Nadu	56.90%	0–3 ppm
Singh et al^34^	2022	Haryana	49.7%	0.19–2.87 ppm
Sharma et al^35^	2023	Haryana	49.10%	Not specifically quantified
Gupta et al^36^	2024	Uttar Pradesh	52.30%	11 ppm
Rani et al^37^	2022	Haryana	51.90%	0.532–8.802 ppm
Punitha et al^9^	2014	Tamil Nadu	19.20%	Gowriammanpettai: 2.1 mg/L, Oliyur-Mottur: 2.0 mg/L, Nallur: 0.4 mg/L, Oliyur: 0.6 mg/L, Vaiyavur: 0.4 mg/L
Manikandan et al^38^	2023	Tamil Nadu	24%	Level I: 56% of sample, level II: 28%, level III: 16%
Rathore et al^39^	2017	Rajasthan	48.00%	<1–>4 ppm
Arya et al^40^	2013	Jammu and Kashmir	76.47%	0.04–4.9 ppm
Rawat and Bafana^41^	2024	Maharashtra	Not numerically specified	1.5–3.6 ppm
Shanthi et al^42^	2014	Andhra Pradesh	74.80%	Stratum 1: <0.7 ppm, Stratum 2: 0.7–1.2 ppm, Stratum 3: 1.3–3.5 ppm
Narwaria and Saksena^43^	2013	Madhya Pradesh	45.46%	1.65–3.91 ppm
Ramesh et al^44^	2017	Tamil Nadu	63.10%	3.7 ppm
Husain and Hussain^45^	2013	Rajasthan	63.60%	0.2–23.2 ppm
Yadav et al^46^	2012	Rajasthan	50%	0.2–14.9 ppm
Gautam et al^47^	2011	Rajasthan	93.12%	0.24–14.62 ppm

**TABLE 3:** Study-Wise Distribution of Fluorosis Risk Contributors

Author	Long-Term Exposure	Lack of Awareness	Fluoride Risk Intake	Socioeconomic Status
Mahantesha et al^11^	−	+	−	−
Sirigala et al^12^	+	−	−	−
Patel et al^13^	−	−	−	−
Sandeep Kumar^8^	+	+	−	−
Choubisa and Choubisa^14^	−	+	+	+
Aggarwal et al^15^	+	−	−	−
García-escobar et al^16^	+	−	−	−
Singh et al^17^	+	−	−	−
Patel et al^18^	+	−	+	−
Sukhabogi et al^19^	+	−	−	+
Verma et al^7^	+	−	+	+
Arif et al^20^	+	+	−	−
Narayanamurthy and Santhuram^21^	+	−	+	+
Naidu et al^22^	+	−	−	+
Chauhan et al^23^	+	−	+	−
Meena et al^24^	−	−	+	+
Ramesh et al^25^	+	−	−	+
Shekar et al^26^	+	+	−	+
Bhalla et al^27^	+	−	−	−
Varsha and Ragini^28^	+	−	−	−
Bhagat P^29^	−	−	+	−
Reddy et al^30^	+	−	+	−
Atram Harish et al^31^	−	−	−	+
Suhirtha and Subramanian^32^	+	−	+	+
Maya et al^33^	−	−	+	−
Singh et al^34^	+	−	+	−
Sharma et al^35^	+	−	−	−
Gupta et al^36^	+	−	+	+
Rani et al^37^	+	−	−	−
Punitha et al^9^	+	−	−	+
Manikandan et al^38^	−	−	+	−
Rathore et al^39^	+	−	−	−
Arya et al^40^	−	+	−	−
Rawat and Bafana^41^	+	+	+	+
Shanthi et al^42^	+	−	+	−
Narwaria and Saksena^43^	+	+	−	+
Ramesh et al^44^	+	−	+	−
Husain and Hussain^45^	+	−	+	−
Yadav et al^46^	−	+	−	+
Gautam et al^47^	−	+	+	+

The table resulting from it gives an overall idea of dental fluorosis prevalence among various Indian states between 2011 and 2024, based on regional studies. Extreme geographic variation is invoked by the data, from a minimum of 4.1% in Himachal Pradesh (Deepak Chauhan, 2012) to a maximum of 94.6% in Uttar Pradesh (Trina Mylena García-Escobar, 2021). Nevertheless, it needs to be noted that the estimates cannot be compared without proper stratification because there are differences in the target population (age range from 7 to 15 years in most cases, although some extended up to adults), design (surveys vs. convenience sampling), sampling strategy (schools vs. communities), and use of diagnostic criteria (the Dean's Fluorosis Index with different cut-off values used). A comparative weighted analysis would have been more desirable in this case; nonetheless, because of differences in the presentation of results and the absence of raw data, a quantitative pooling could not be performed in the present study. Very high prevalence was also reported in the states of Gujarat (93.7%), Maharashtra (up to 79.2%), Andhra Pradesh (up to 82.04%), and Rajasthan (study variation with over 60%). These differences are exactly mirrored by the varying fluoride content in natural sources of water, ranging from a minimum of 0.04 ppm to a maximum of 23.2 ppm, far more than the WHO-laid upper limit of 1.5 ppm in most of these cases.

A few of the research studies, such as Shanti Lal Choubisa and IKBAL HUSAIN, have reported the very high levels of fluoride (21.6 and 23.2 ppm, respectively) to which the respective high levels of the incidence of fluorosis are due. Regions with a relatively lower level of fluoride, e.g., Chhattisgarh districts (Bhagat P., 2019; 0.5–6 ppm), also had their occurrences, though at a lower rate (∼14.3%). Many of the studies, for example, by Charu Aggarwal and Ashish Bhalla, were not quantitative in their reporting of fluoride content, lowering the quality of correlation studies. Tamil Nadu and Andhra Pradesh were 2 of the best-studied states, a reflection of intraregional heterogeneity because of heterogeneity in water sources (government supply vs. groundwater) and aquifer depth. The results emphasize the unprecedented requirement for geographical region-specific water quality surveillance and focused public health interventions to reduce the risk of fluorosis.

Table 2 provides a comprehensive comparative analysis of 40 peer-reviewed articles carried out between 2010 and 2024 in different Indian states, evaluating the prevalence of 4 significant risk factors for dental fluorosis: long-term exposure to fluoride, unawareness, overconsumption of high fluoride content, and economic status. The table follows a binary indicator format where a plus sign (+) signifies that the mentioned study mentioned and commented on the aforementioned risk factor as a causative agent of dental fluorosis, and a minus sign (−) indicates that the aforementioned study did not report or comment on that parameter. The graphical presentation helps to know how often and in what manner these factors are recognized through studies and regions.

Long-term exposure consists of long-term drinking of water with excessive fluoride, usually for many years, and is more likely to cause fluorosis. Recurring exposure causes most harm during the period of tooth development (first 8 years of life) because additional fluoride gets deposited into enamel, causing hypomineralization and detectable dental alterations. Studies highlighted here in this column with the (+) symbol underscore the acute nature of fluoride poisoning, particularly in the rural population relying on deep borewells or unregulated groundwaters.^3^

The unawareness pertains to the knowledge deficit of the population in terms of the source of fluoride, symptoms, effects of fluorosis, and prevention or remedial action. (+) Here signifies that ignorance among the population was one key constraint to early prevention and diagnosis of fluorosis, as supported by the study. Most studies, particularly from the endemic areas, illustrate how people with fluorosis are likely to confuse signs of fluorosis with normal discoloration or staining and therefore seek late treatment.

Fluoride-rich intake indicates consumption of foodstuffs or cultural products containing naturally or industrially high fluoride content. These include the tea (particularly brick tea), gutkha, and tobacco items, and agricultural products such as ragi (finger millet), which both contain fluoride from the soil and water. (+) This column indicates that the survey was also adjusted for such lifestyle or food factors of a higher fluoride burden. Observe that these medicines are individually more likely to be consumed by lower-income or rural communities, imposing extra risk. Socioeconomic status (SES) with (+) indicates that the study population was mostly from low-income families. Low SES is a known indirect risk factor because individuals in such environments are apt to use fluoridated groundwater because of the lack of a piped or treated water supply, possess poor access to dental care and health education, and are less likely to be able to afford nutritional diets that can counteract fluoride toxicity. These groups are also less likely to demand infrastructure changes and, therefore, more susceptible to long-term health damage such as skeletal and dental fluorosis. From the table, one can see that various studies have come to a variety of coinciding factors to be responsible for fluorosis, as opposed to one.

For instance, Andhra Pradesh, Rajasthan, and Maharashtra all reported all 4 risk factors to contribute, showing how combined vulnerabilities create a greater prevalence. At the same time, some research analyzed fluoride in water only without explaining socioeconomic or behavioral determinants, expecting research gaps to be filled to provide an integrated picture. This table is not just useful to synthesize literature but also in informing public health planning, programmatic interventions, and research directions. It demonstrates that dental fluorosis in India is not just an environmental or chemical problem but also an educational, dietary, and social problem requiring solutions that are multifaceted to tackle.

### Risk of bias profile of included dental fluorosis studies based on Cochrane criteria

This figure illustrates the risk of bias assessment in 7 methodological areas commonly evaluated in research studies. Green bars represent a low risk of bias, yellow (not depicted herein) represents an uncertain risk, and red represents a high risk of bias. For selection bias in the generation of random sequence, most of the studies were low risk, but a few with high risk since there were some studies with inadequate randomization methods. Allocation concealment was also mostly of low risk, but a slightly higher proportion was of high risk, which is indicative of potential deficits in protection against selection bias in participant allocation. Participant and staff blinding (performance bias) and outcome assessment blinding (detection bias) were low risk in the majority of studies, with a few that did not blind participants, staff, or assessors. Reporting (selective reporting or reporting bias) and incomplete outcome (attrition bias) were low risk in most of the studies, but some reported high risk because of most likely missing outcomes or missing data. Finally, other bias was also overall low risk, with few high-risk cases, and suggest most studies did not include important supplementary methodological weaknesses. Overall, although the vast majority of studies were of good methodology, there was a minority of important weaknesses, especially in blinding and allocation concealment (Figure 8).

**FIGURE 8:**
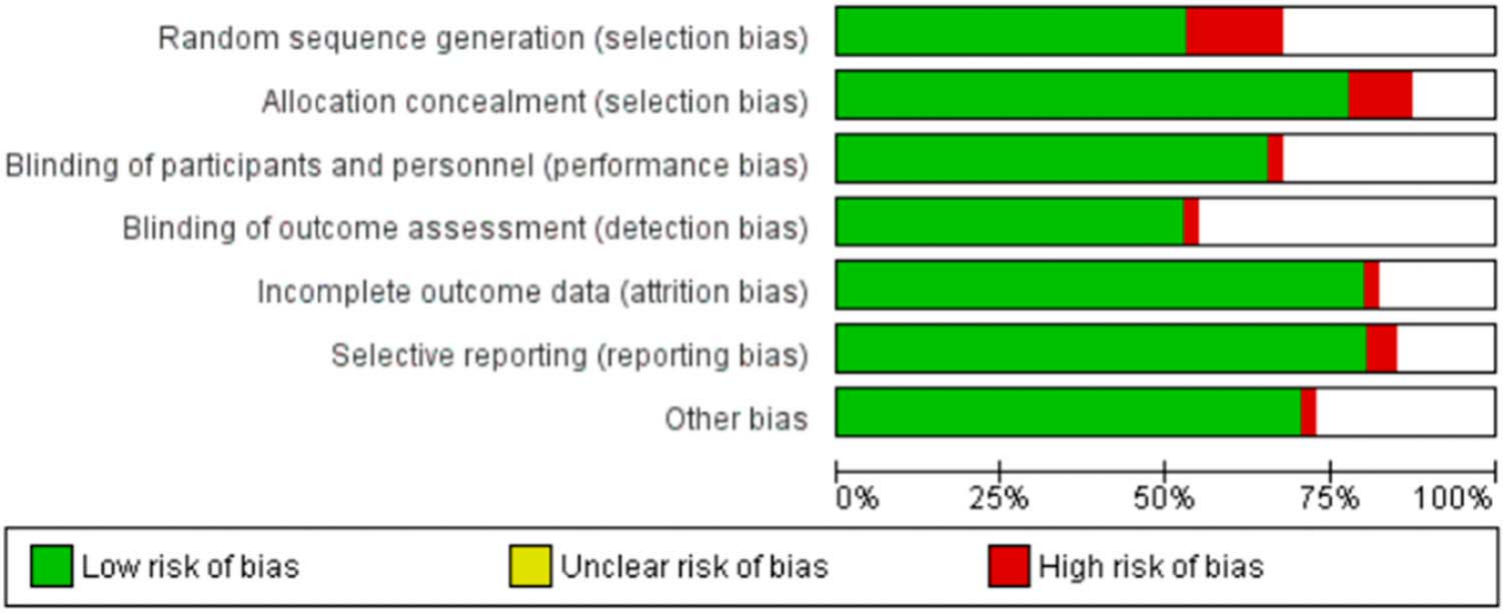
Risk of bias profile of included dental fluorosis studies.

## Discussion

This scientometric analysis offers a general overview of patterns in dental fluorosis research in India in volume of publications, thematic focus, geographic location, and cooperative networks. The record is evident in monolithic spatial imbalance, with a compact group of studies proceeding from fluoride-endemic states such as Andhra Pradesh, Tamil Nadu, Maharashtra, and Karnataka, and under-representation states such as Odisha, Bihar, Jharkhand, and the Northeastern states, although there is likely fluoride exposure in rural communities. This geographical bias could be because of both a lack of infrastructure to track fluoride, along with the absence of available resources for academia in the regions, compromising blind spots in the country's surveillance. Chronologically, the pattern of publication activity is not even, with peaks observed during 2013, 2017, and a sharp rise once more in 2023. This diverse production informs us that dental fluorosis is an episodically readdressed but not persistently sequential research topic, and thus has ongoing scientific interest and priority focus awaiting.

The co-author network analysis describes modestly cooperative sets of studies with some notable contributors, such as Maya Ramesh, as focal points of their networks. However, there are no inter-regional or pan-Indian collaborations, which suggests compartmentalization of research effort as opposed to a concerted, national effort. Co-occurrence keyword mapping illustrates salient themes of “prevalence,” “fluoride,” “dental fluorosis,” and “children,” indicating extreme reliance on cross-sectional and descriptive epidemiologic research designs. However, new phrases such as “urinary fluoride,” “skeletal fluorosis,” and “rural population” indicate widening horizons.

Surprisingly, studies on behavioral, dietetic, or socioeconomic etiologies of fluorosis remain limited. This incompatibility is critical because some of the high-burden areas are also poor, low-literacy, and reliant on fluoride-contaminated food sources like tea, ragi, and tobacco. Similarly, community education and health education, both key to prevention in the longer term, are rarely studied or systematically reported. This lack of thorough assessment limits the translational utility of current evidence and reduces its policy relevance. The findings of this study have implications for surveillance strategy, water safety management and the level of intervention within the community and for health policy. First, regarding surveillance, there is a huge gap in the surveillance of fluoride-related problems in states like Bihar, Odisha, West Bengal, Jharkhand, and all over the Northeastern region of the country that warrants the need for systematic fluoride-related surveillance that is not only academic but institutional. The National Programme for Prevention and Control of Fluorosis (NPPCF) needs to be used to ensure population representative dental fluorosis surveys have taken place in under researched districts and that reporting formats are standardized so that future pooled analyses are possible. The cost and logistics of fluorosis screening would also be minimized if screening of fluorosis was built into activities of the National Oral Health Programme. Second, the high prevalence of groundwater fluoride levels exceeding the WHO guideline (1.5 mg/L) in the endemic states indicates a need for augmentation of defluoridation programs to improve water safety management. The water quality parameters collected from the literature survey can be used to prioritize the districts in the need of defluoridation infrastructure by the state governments and CGWB. Third, as per the community level, most of the reported risk factors by different studies are unawareness which again indicates the need for specific health education campaigns. Oral health education programs in schools, ASHA worker training in identification of fluorosis should be linked with Rashtriya Bal Swasthya Karyakram (RBSK) and school health component of Ayushman Bharat, especially at the community level in rural and tribal areas. Fourth, regarding policy implications and capacity building, the author network is more concentrated in the southern and western institutions. Fourth, in the policy implications and capacity building part, the southern and western institutions have a more focused author network of the dental public health area. There should be incentives from National institutions like Dental Council of India (DCI) and Indian Dental Association (IDA) to the postgraduation Dissertation research on Fluorosis in states with low level of research and ICMR should consider the provision of targeted Extramural grants on Fluorosis epidemiology in areas with low research activity. These network disconnections identified in the co-authorship analysis could be resolved by the formation of multi-institutional research consortia, which would enable inter-regional collaboration, especially between endemic states in the north and the northeast. The multifaceted and socially embedded nature of dental fluorosis in India is also reflected on the need for coordinated efforts in implementing the evidence into programs: translating scientific evidence into actionable program implementation requires concerted efforts across health, water, and education and environmental sectors.

Students' and researchers' familiarity with various bibliometric software programs will support scientometrics research more effectively.^48^ The programs used in this article were more familiar and easily accessible, which gives data with precision and accuracy. A literature review suggests that fluoridated water, fluoride supplements, infant formula, and fluoride toothpaste are risk factors for fluorosis, and the risk of developing fluorosis increases with the use of these products.^49^ Similar to the above-mentioned phrase, the fluoride risk contributor analysis in this article shows that fluoride-rich intake has more influence on increasing dental fluorosis. In a meta-analysis conducted by Shakir Ali et al^50^ to analyze the water fluorosis levels across India, they found that the combined fluoride concentration in India was around 2.37 mg/L (95% CI: 1.46–3.28 mg/L). Because this range is much above the 1.5 mg/L WHO-recommended limit of safety, unsafe fluoride levels in drinking water are likely to occur in most regions of India and may result in extensive skeletal and dental fluorosis, which relates to the geographic distribution explained in this study.

In a study conducted in Mexico by Nelly Molina-Frechero et al,^51^ Youths in the medium-SES group had less severe dental fluorosis, accounting for the lowest TF values and having fewer individuals with high severity, whereas those in the low-SES group tended to be more concerned about their teeth. These differences based on SES were significant. By contrast, a study conducted in the Alappuzha district, Kerala, by P. Gopalakrishnan et al^52^ states that socioeconomic status does not influence dental fluorosis in their study area. But from this study, the analysis explains that the majority of studies do not consider socioeconomic status as a risk factor.

## Conclusion

This scientometric synopsis accounts for the rising but uneven base of dental fluorosis research in India. There is a high concentration of studies in southern and western states, while fluoride-endemic northern and northeastern states are relatively undercovered. Studies take an upward trajectory of output spanning years, reflective of growing concern for the issue. Keyword mapping reveals a strong focus on prevalence, severity, and clinical presentation. Fewer papers focus on socioeconomic determinants, fluoride-rich intake, and water fluoride levels. Co-authorship networks reveal little institution-based and interregional collaboration. This study serves as the foundation for determining the gaps in the area of research and guiding subsequent studies in helping to reduce India's prevalence of fluorosis.

## Limitations

This scientometric review is constrained by the literature scope and availability for inclusion in the databases; some relevant regional research may have been missed because of database limitations. Discrepancies in study design, reporting, and methods for fluoride assessment between the included publications could have influenced the study's comparability of prevalence data and risk factor data. In addition, there could be language bias since only English-language publications were considered.

**Implications for Policy & Practice**

■ Strengthen surveillance and research in underserved regions. National and state health authorities should prioritize funding for epidemiological surveillance and research in fluorosis-endemic but underrepresented states. A geographically balanced evidence base is essential for equitable fluoride mitigation strategies and effective resource allocation.
■ Regular monitoring of drinking water fluoride levels should be incorporated into existing public health and primary healthcare programs, alongside community-based oral health education, early screening, and defluoridation initiatives in high-risk areas. Such integration would facilitate early detection and reduce the long-term burden of dental fluorosis.
■ Policymakers and funding agencies should encourage inter-institutional collaborations among dental schools, public health departments, environmental agencies, and water authorities. Future research should extend beyond prevalence studies to include behavioral, socioeconomic, environmental, implementation, and policy-focused investigations that can directly inform national fluorosis prevention and control programs.
